# miRNAtools: Advanced Training Using the miRNA Web of Knowledge

**DOI:** 10.3390/ncrna4010005

**Published:** 2018-02-16

**Authors:** Ewa Ł. Stępień, Marina C. Costa, Francisco J. Enguita

**Affiliations:** 1 Department of Medical Physics, Marian Smoluchowski Institute of Physics, Jagiellonian University, 30-348 Kraków, Poland; e.stepien@uj.edu.pl; 2 Instituto de Medicina Molecular, Faculdade de Medicina, Universidade de Lisboa, Av. Professor Egas Moniz, 1649-028 Lisbon, Portugal; marinacosta@medicina.ulisboa.pt

**Keywords:** miRNA, web tools, advanced teaching, tutorials

## Abstract

Micro-RNAs (miRNAs) are small non-coding RNAs that act as negative regulators of the genomic output. Their intrinsic importance within cell biology and human disease is well known. Their mechanism of action based on the base pairing binding to their cognate targets have helped the development not only of many computer applications for the prediction of miRNA target recognition but also of specific applications for functional assessment and analysis. Learning about miRNA function requires practical training in the use of specific computer and web-based applications that are complementary to wet-lab studies. In order to guide the learning process about miRNAs, we have created miRNAtools (http://mirnatools.eu), a web repository of miRNA tools and tutorials. This article compiles tools with which miRNAs and their regulatory action can be analyzed and that function to collect and organize information dispersed on the web. The miRNAtools website contains a collection of tutorials that can be used by students and tutors engaged in advanced training courses. The tutorials engage in analyses of the functions of selected miRNAs, starting with their nomenclature and genomic localization and finishing with their involvement in specific cellular functions.

## 1. Introduction

Micro-RNAs (miRNAs) are small non-coding RNAs (ncRNAs), firstly described in the early 1990s by Victor Ambros, Gary Ruvkun, and their respective research groups [1,2]. These tiny ncRNAs associated with specific proteins act as negative post-transcriptional regulators by binding to the 3′-untranslated region (3′-UTR) of messenger RNA (mRNA) transcripts. The miRNA binding to the 3′-UTR of a mRNAs is based on their complementarity and ensured by the Watson–Crick base pairing rules. In animals, the miRNA is only partially complementary to its mRNA target, typically involving nucleotides 2–9 of its 5′ end (seed sequence). However, in plants, the complementarity of the miRNA and its target is typically higher than 90%. These different pairing rules in animals and plants are translated in different immediate regulatory effects. Whereas in animals the partial complementarity produces a translational repression as a main regulatory effect [3], the almost complete complementarity between miRNAs and their targets observed in plants ensures a degradation of the target mRNA as a primary consequence [4,5].

miRNAs are global regulators of cellular pathways and constitute a layer of post-transcriptional control that is superimposed on classical transcriptional regulators. The small size of miRNAs is responsible for their regulatory promiscuity: a single miRNA can regulate hundreds of different mRNA transcripts; at the same time, a given mRNA transcript can be targeted by several miRNAs. However, the regulatory effect exerted by a miRNA is only possible if the miRNA and its cognate target are present simultaneously in the same biological environment. This is the subjacent reason below the variety of regulatory effects of the same miRNA when we consider a different cellular context.

Since the discovery of miRNAs, many bioinformatics tools have been developed with the objective of studying their physiological roles [6,7,8]. Among the bioinformatics applications related to miRNAs, target prediction tools are the most important and the basis for the majority of the existing tools for miRNA functional analysis [9,10,11]. Target prediction algorithms have evolved over time, from classical ones that only took the base complementarity roles between miRNAs and their targets into account [3,6] to modern ones based on deep learning and neural network algorithms [12,13]. Other computer applications take advantage of target prediction algorithms to infer the physiological roles for miRNAs [14,15,16]. The evolution of scientific protocols has also dramatically changed the requirements of computer applications related to miRNAs. For instance, some of the original applications designed for the prediction of miRNA genes across genomes have become obsolete with the introduction of next-generation sequencing techniques [17,18]. In fact, the web landscape is densely populated with web tools designed for miRNA analysis, which in many cases are not so easy to find or to use.

## 2. Results

During the last six years, we have been involved in advanced teaching initiatives for students in biomedical sciences ranging from the post-graduate to the post-doctoral level within the field of miRNAs and other ncRNAs. We have organized lecture and practical courses and workshops in our institution and abroad to introduce the field of miRNAs in a practical manner using bioinformatics tools. From the very beginning, it was evident to us that students faced a problem: there is a myriad of bioinformatics tools dispersed on the web, and they are not always easy to use or interpret. Moreover, the fast evolution of laboratory analytical techniques and computer software very quickly rendered some of these tools obsolete. Some excellent compilations of miRNA web tools such as Tools4miRs [19], miRandb [20], and miRtoolsGallery (http://mirtoolsgallery.org/miRToolsGallery) have been designed with the main objective of systematizing, classifying, and organizing all of the available web tools within the field of miRNAs. However, there was a clear need for specialized teaching material devoted to the study of miRNAs and other ncRNAs that could be employed as self-teaching tools and as conducting scripts for advanced courses. Thus, we created miRNAtools (http://mirnatools.eu), a repository of web tools devoted to the study of miRNAs, which is complemented by specialized tutorials for assisted or self-learning. The main difference between miRNAtools and all other existing repositories of web tools is its specific design for advanced teaching.

### 2.1. Structure of the miRNAtools3 Website

**Figure 1:**
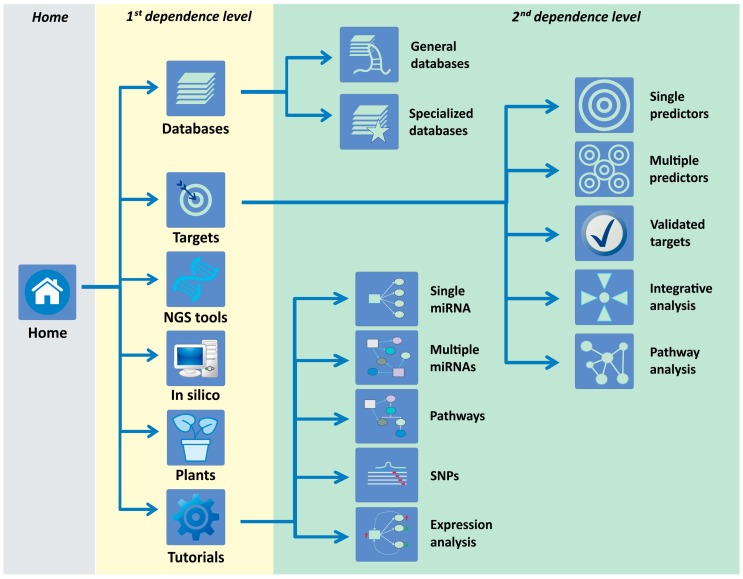
Navigation structure of the miRNAtools3 website showing the two main dependence levels. Navigation across sections and topics can be achieved by direct pointing over the respective icons or by using the navigation menu integrated in each sub-page.

The miRNAtools website (http://mirnatools.eu) has evolved from a preliminary β version launched in 2010 to the new miRNAtools3, which has been available on the web since May 2017. miRNAtools3 is built in HTML 5.0 and complemented with cascading style sheets (CSS) and Javascript aids that allow for rapid and easy navigation between sections. The overall flexible web design ensures a rapid update of sections and the inclusion of new topics, tutorials, and applications without changing the general outlook. The tutorial structure of the webserver has been served as a basis for practical training in seven specialized courses about ncRNA biology organized in the University of Lisbon (Lisbon, Portugal), the Universidade Estadual Paulista (UNESP) (São Paulo, Brazil) and the Jagiellonian University (Krakow, Poland) and used directly by more than 250 students as participants in our courses. The number of external users of the website, as quantified by the page accesses, is close to 500. The miRNAtools3 website contains two dependence levels (Figure 1): The first one is currently structured in six main sections: databases, targets, next-generation sequencing (NGS) tools, in silico tools, plant miRNAs, and tutorials. The “Databases” section contains two sub-sections compiling “General purpose databases,” compiling links to miRNA-related databases for general use, and “Specialized databases” that contain databases designed for studying specific sub-fields of the miRNA world, such as circulating miRNAs, the evolution and function of miRNAs, and disease-related databases.

The “Targets” first dependence level is the most complex and includes five sub-sections: single predictors, multiple predictors, validated targets, integrative analysis, and pathway analysis. The “Single predictors” sub-section is devoted to compile web applications for miRNA target prediction. Every link to an external website is accompanied by a personal assessment of the advantages and disadvantages of each algorithm and the corresponding link to a PubMed (National Institutes of Health, Bethesda, MD, USA) reference. The “Multiple predictors” second dependence level includes web-based applications able to interrogate multiple target predictors at the same time. These applications are especially powerful since they are able to generate a stronger and more reliable target prediction than the individual algorithms considered alone. The “Validated targets” sub-section includes web tools and databases for the retrieval of validated miRNA targets. The last two sub-sections within the “Targets” first dependence level are devoted to the functional analysis of miRNA action. The “Integrative analysis” contains web tools designed for the integration of transcriptomic data and miRNA expression, and the “Pathway analysis” sub-section is composed of bioinformatics tools that infer the action of one or several miRNAs over cell metabolic pathways. Every item within sections and sub-sections contains a personal overview and description and a reference to its publication. The first dependence level of miRNAtools3 is completed by three additional independent sections covering tools related with miRNA target analysis in plants: in silico tools for the prediction of novel miRNA genes in new genomes, tools for NGS analysis of miRNA expression, and tutorials.

### 2.2. Tutorials within miRNAtools3

**Figure 2:**
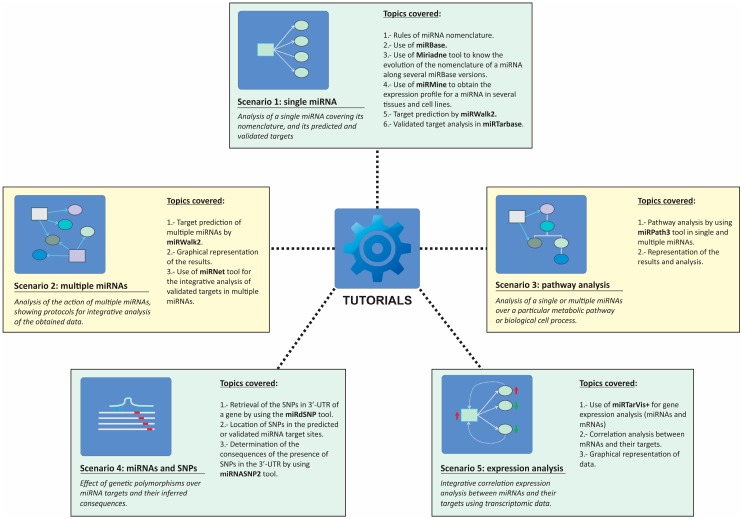
Structure of the tutorials section of miRNAtools3 showing all the proposed working scenarios and the topics and computer applications covered by each of them.

The tutorial section is designed for self-teaching but also serves as a main guiding script for specialized courses or workshops. This section is divided into proposed scenarios that introduce a biological example involving a particular miRNA or groups of miRNAs and guide the user through different web applications to obtain relevant information about the biological problem. All the scenarios are explained by using screenshots of the selected web tools in order to guide the student in terms of the inputs and outputs of every application. The specific applications and topics covered in each tutorial are depicted in Figure 2.

#### 2.2.1. Scenario 1: Single miRNA

The proposed scenario used a real example published by Asangani et al. in 2008 [21] about an oncogenic miRNA, miR-21. The students are guided along the tutorial; students start with simple steps such as the retrieval of relevant data about the selected miRNA in miRBase [22,23] and learn about proper miRNA nomenclature. The tutorial also shows how one converts old-style miRNA nomenclature used in old releases of miRBase into nomenclature used in miRBase v.18. In order to facilitate the nomenclature inter-conversion between miRBase versions, the tutorial explains how one can utilize web tools such as miRiadne to follow the evolution of different nomenclatures for miR-21 along all miRBase versions [24]. This step is relevant for those wishing to conjugate information about a particular miRNA that can be found in recent and old literature.

Students will also learn how to retrieve expression data for the selected miRNA in different human cells and tissues by the use of miRmine, a recent database which compiles expression profile for miRNA in human tissues [15]. The final part of the tutorial focuses on the retrieval of information about predicted and validated targets for the selected miRNA, hsa-miR-21-5p. For the prediction of miRNA targets, the miRNAtools3 users are encouraged to use multiple predictors such as miRWalk2, since these applications are able to simultaneously interrogate several target prediction algorithms, displaying results in a graphical manner and allowing the user to select those targets predicted by a higher number of algorithms [10]. For the retrieval of validated targets, the users are guided into the use of miRTarBase, a recently updated database of validated miRNA targets [9].

#### 2.2.2. Scenario 2: Multiple miRNAs

For the task of analyzing the simultaneous action of several miRNAs, we have proposed studying the targets of the miR-17-92a cluster, which is known to be overexpressed in some human tumors as lymphomas and involved in cell proliferation [25]. The main objective of this tutorial is to obtain information about the putative targets for the components of the miR-17-92a cluster, highlighting which of those targets could be more physiologically relevant. Based on a circular layout graphical representation of the miRNA–target relationships, we proposed employing a strategy described in [26] that will allow for a determination of which mRNAs are simultaneously targeted by a higher number of miRNAs and which miRNAs are able to regulate more targets. We always encourage students to employ graphical depictions of results obtained from target prediction algorithms, since, compared with a tabular format, such representations are easier to interpret and allow more information to be displayed. The tutorial explains how these interaction networks are constructed using a manual approach in the case of predicted targets or using an automatic application for validated targets as described in MirNet [11].

#### 2.2.3. Scenario 3: Pathway Analysis

Based on our teaching experience, students are often more stimulated when they can infer physiological implications of their data. Scenario 3 was designed with the objective of making functional sense of the target prediction algorithms for a group of miRNAs. As in Scenario 2, we employed the human miR-17-92a cluster as an example, but in this case the tutorial guides the user in the prediction of the putative cellular pathways regulated by this six-membered cluster. Among the existing web tools used to perform miRNA pathway analysis, we selected a module of the DIANA-microT prediction suite, DIANA-miRPath v3.0, mainly because of its easy-to-use web interface and graphical output [16]. The tutorial teaches the user how to perform a pathway and GO-term enrichment analysis of the predicted and validated targets of the cluster members by using miRPath3 and how to produce graphical representations of the results. The tutorial explains also how filters are applied to the target information by relevance scores and explains the meaning of these scores in the context of a cellular pathway, thereby helping users provide productive and meaningful interpretations of obtained results.

#### 2.2.4. Scenario 4: Single Nucleotide Polymorphisms and miRNA Binding Sites

This scenario is an interesting example of how the miRNA action can be considered within the field of pharmacogenomics. The proposed scenario is supported by a Genomic Wide Association Study (GWAS) published by Zuo et al. [27], who described some genetic variants in the gene encoding for the cannabinoid receptor (CNR1) that might be related to an increased risk for cocaine dependency. In fact, one of these variants named rs806368 is located in the 3′-UTR of the *CNR1* gene. The tutorial describes how information about the location of this single nucleotide polymorphisms (SNP) variant is found and describes the influence that this variant can eventually have in the putative binding of specific miRNAs. During the tutorial, the user is trained to use two databases: miRdSNP [28], a database of human disease-associated SNPs and miRNA targets, and miRNASNP2 [29], a compilation of genotypic variants extracted from GWAS studies and related to miRNA binding sites. At the end of the tutorial, the user should be able to analyze the effect of a particular genetic variant over the binding of a miRNA, by calculating the free energy of the miRNA–mRNA hybrid in the presence and in the absence of the genetic variant.

#### 2.2.5. Scenario 5: miRNA–mRNA Expression Correlation Analysis

The biological action of a particular miRNA depends on the simultaneous existence of its cognate mRNA target(s) in the same cellular context. The characterization of the biological roles of miRNAs is often complex since cells are extremely redundant. Frequently, transcriptomic analysis is used as starting point to determine the relevance of the action of miRNAs over the cell metabolism [30]. This tutorial describes how gene expression data can be used to characterize the putative action of a group of miRNAs via correlation analysis. In a biological system, an inverse correlation of expression levels between a miRNA and its cognate target is expected, but whether this effect is evident at the level of mRNA transcript depends on the specific characteristics of the considered mRNA [31,32]. In this tutorial, we describe the use of a web-based tool named MirTarVis+ [14,33], which is able to perform such a correlation analysis, with results depicted in a graphical manner. The tutorial is based on real expression data collected by microarray analysis in tumor and normal cells. The corresponding data files are supplied to the user, and the specific format for input files in MirTarVis+ is explained. The tutorial follows the logical flowchart of MirTarVis+, starting with data filtering and the inverse correlation analysis of miRNAs and their targets and finishing with a graphical representation of the results that allow the user to determine which miRNAs and targets might be more relevant for the selected biological process.

## 3. Conclusions and Further Perspectives

Advanced teaching initiatives for post-graduate students are essential for their integration into scientific culture and their exposure to scientific methodology, especially in areas where there is a rapid accumulation of scientific knowledge. We have designed a flexible web tool specifically designed to teach miRNA function using only web-based applications, and we believe that this took satisfies an unmet need in this particular field. Our web tool has been extensively improved by its inclusion of tutorials given to students within workshops and courses dedicated to the study of miRNAs and other ncRNAs that have been taught in our institution and abroad. Further developments of miRNAtools will be ensured by its intrinsic flexibility. The expansion of the number of proposed scenarios for teaching will take into account the valuable input received from students and users on the website. Moreover, we are working in similar web repositories for the study of long non-coding RNAs (lncRNAs) and circular RNAs (circRNAs), which will both be incorporated into miRNAtools.
